# Neuroform atlas stent treatment for 533 intracranial aneurysms in a large Chinese cohort: complication risk factor analysis

**DOI:** 10.1186/s12883-024-03695-z

**Published:** 2024-06-10

**Authors:** Shibao Chen, Huibin Kang, Dili Wang, Yan Li, Jamali Aikebaier, Yabo Li, Xinshan Wu, Yuhua Guan, Yisen Zhang

**Affiliations:** 1 Department of Neurology, People’s Hospital of Bayingoleng Mongolia Autonomous Prefecture, No. 41 Renmin East Road, Korla, 841000 Xinjiang China; 2 Department of Neurosurgery, Nanfang Hospital, Southern Medical University, Guangzhou, China; 3 People’s Hospital of Jiangxia District, Wuhan, China; 4 Third People’s Hospital of Jinan, Jinan, China; 5 Department of Interventional Neuroradiology, Fengtai District, Neurosurgical Institute and Beijing Tiantan Hospital, Capital Medical University, No. 119 South Fourth Ring West Road, Beijing, 100050 China

**Keywords:** Intracranial Aneurysms, Neuroform Atlas Stent, Treatment, Complication, Risk Factor

## Abstract

**Background:**

The newest generation of Neuroform Atlas stent™ (Stryker, Fremont, California) represents a recent advance of cerebral laser-cut microstents for the treatment of intracranial wide-necked aneurysms, and postoperative complications have been observed among Western patients. We assessed predictors of complications, morbidity, and unfavourable outcomes in a large cohort of patients with aneurysms that were treated with Neuroform Atlas stents in China.

**Methods:**

This retrospective study included subjects who were treated with Atlas stents in China from November 2020 to January 2022.

**Results:**

A total of 522 consecutive patients (mean age, 58.9 ± 9.9 years; female, 65.3% [341/522]) with 533 aneurysms were included in the study. In the early postoperative period, the neurological morbidity rate was 7.3% (38/522), the ischaemic stroke rate was 5.0% (26/522), the aneurysm rupture subarachnoid haemorrhage rate was 2.3% (12/522), and the mRS score deterioration rate was 5.4% (28/522). The mortality rate was 0.8% (4/522) in the postoperative period. The rate of neurological morbidity during the follow-up period was 1.2% (6/486). In the multifactor prediction analysis, cerebral infarction, Hunt–Hess grade (3–5), procedure duration, stent length and coil protrusion into the parent artery were found to be independent predictors of neurologic morbidity. The procedure duration, stent length and coil protrusion into the parent artery were found to be independent predictors of mRS score deterioration.

**Conclusions:**

The incidence of SCA (stent-assisted coiling)-related complications with the Atlas stent in this study population was comparable to that in Western populations. We identified the procedure duration and stent length as novel independent predictors of SCA-related ischaemic stroke, neurological morbidity, and mRS score deterioration among the Chinese population.

**Supplementary Information:**

The online version contains supplementary material available at 10.1186/s12883-024-03695-z.

## Background

The prevalence of intracranial aneurysms in China is 7.0%, and these aneurysms are the leading cause of nontraumatic subarachnoid haemorrhage [1]. The treatment of intracranial aneurysms using SAC has been practised for more than 20 years. Compared to coiling alone, SAC has shown better occlusion rates, and flow diversion has an occlusion rate of up to 80% with an acceptable safety profile [2–4]. One study reported that a deep learning system can improve the surgical planning of coil embolization for UIAs [5]. However, compared with coiling alone or balloon-assisted coiling, stent-assisted coiling is associated with a higher complication rate. A recent meta-analysis of low-profile stents showed a procedure-related complication rate of 12.4% [6]. At present, many studies have been published on procedure-related complications associated with the use of intracranial stents, such as Neuroform (Stryker, Kalamazoo, MI, USA), Enterprise (Codman, Raynham, MA, USA), Solitaire (Covidien, Irvine, CA, USA) and LVIS (MicroVention, Tustin, California)[7]. Some recent studies have reported the safety and effectiveness of Atlas stent implantation for the treatment of intracranial aneurysms [8–10]. Due to the small sample size, these studies have mainly focused on statistical analyses of the occlusion and complication rates and lacked detailed analyses of postoperative complication risk factors, and most of the studies have involved Western populations. Importantly, Chinese populations differ from Western populations in terms of the prevalence of comorbidities such as intracerebral haemorrhage (ICH) and intracranial atherosclerosis, which may influence the risk of ischaemic stroke [11, 12]. Thus, the purpose of this study was to assess the predictors of complications and functional outcomes in Chinese patients who underwent Atlas stent-assisted coiling for the embolization of intracranial aneurysms. To our knowledge, this is the largest retrospective cohort study of procedure-related complications with the recently introduced small Atlas stent.

## Methods

### Ethics approval, participants and study design

We retrospectively evaluated the clinical data of 522 patients with 533 intracranial aneurysms, including both ruptured and unruptured aneurysms, who underwent Neuroform Atlas stent implantation between November 2020 and January 2022 in China. The study was approved by the medical ethics committee of Beijing Tiantan Hospital. The requirement for informed consent was waived due to the retrospective design of the study.

Patient demographic data, including sex, age, hypertension status, diabetes status, hyperlipidaemia status, cerebral infarction status, cardiac disease status, smoking status, alcohol abuse status, presentation status, Hunt–Hess grade, previous treatment status, CYP2C19 genotyping, platelet aggregation test (PAgT) results, and collagen arachidonic acid (AA) and adenosine diphosphate (ADP) levels, were collected by reviewing the medical charts.

The following aneurysm characteristics were extracted from digital subtraction angiography (DSA): aneurysm size, aneurysm neck size, aneurysm width, aneurysm height, parent artery diameter, size ratio (SR), aspect ratio (AR), height/width ratio (HW), aneurysm form, and aneurysm location. Aneurysm size is most commonly defined as the “maximal dimension of the aneurysmal dome” in the literature[13, 14]. Neck size is defined as the maximum neck diameter, aneurysm width is defined as the maximal longitudinal diameter of the dome parallel to the neck plane, aneurysm height is defined as the maximum neck diameter, SR is defined as the maximal aneurysm height/parent vessel mean diameter, AR is defined as the maximal distance from the neck to the aneurysm dome/maximal neck width, and HW is defined as the maximal distance from the neck to the aneurysm dome/maximal width of the dome orthogonal to the maximal height. Aneurysm form was categorized as saccular or nonsaccular. Aneurysm location was specified as anterior or posterior circulation. The anterior circulation was divided into proximal and distal anterior circulation. The proximal anterior circulation spanned from origin of the internal carotid artery to the bifurcation of the internal carotid artery, including the ophthalmic artery, arterial segment in the cavernous sinus, posterior communicating artery, superior hypophyseal artery and posterior carotid wall. The distal anterior circulation included the arteries branching from the internal carotid artery, including the anterior and middle cerebral arteries [15]. The posterior circulation was divided into the vertebral-basilar and other vessels in the posterior circulation (the posterior cerebral artery, posterior inferior cerebellar artery, anterior inferior cerebellar artery and superior cerebellar artery).

The study endpoints included functional outcomes (modified Rankin scale [mRS] score) and complications related to AS treatment in the early postoperative period (< 30 days) and at the last clinical follow-up (3 to 17 months, mean 9.7 months). Multivariate analysis was performed to identify risk factors for complications.

### Procedural details

Sixteen interventional neuroradiologists performed the stenting procedure in this study. Electively treated patients took 75 mg of clopidogrel and 100 mg of aspirin daily for at least five days before the procedure. A plate aggregation test and CYP2C19 genotyping test were performed to ensure a good response to clopidogrel. Patients were switched from clopidogrel to ticagrelor (loading dose of 180 mg and daily maintenance dose of 90 mg twice daily) if they were hypo-responders to clopidogrel. All patients were prescribed 75 mg of clopidogrel daily for 12 weeks and 100 mg of aspirin daily for at least 12 months after the procedure. All procedures were performed under general anaesthesia, and a glycoprotein (Gp) IIb/IIIa inhibitor (tirofiban) or heparin was administered intravenously during the procedure, before or after stent release; the dosage was determined by the treating physician. Furthermore, the flush system contained heparin at an I.E. of 3000 per litre. After the coil was inserted into the aneurysm cavity, a bolus of 3000 IU of heparin was infused before or after the stent was deployed. Subsequently, 1000 IU of heparin was infused per hour during the procedure. If acute thrombosis occurred or coil herniation to the parent artery occurred during the procedure, a glycoprotein (Gp) IIb/IIIa inhibitor (tirofiban) was used. The dosage and duration were determined by the treating physician.

### Patient follow-up and end points

**Fig. 1:**
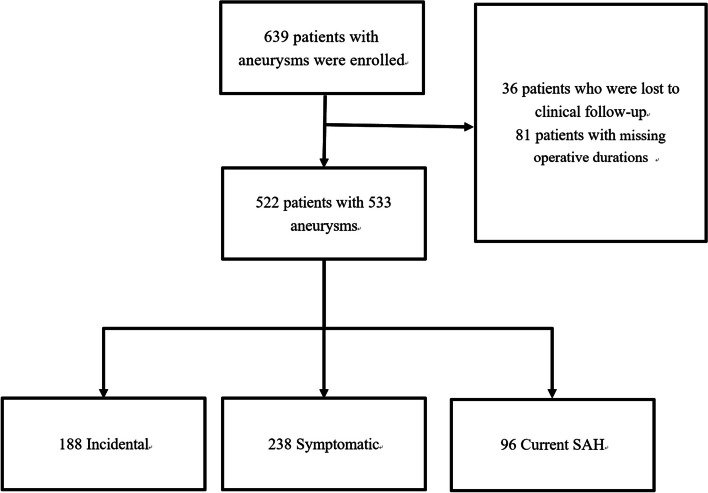
Flow chart of patient selection and grouping

The flowchart is shown in Fig. 1.Patients were scheduled for clinical follow-up at intervals of 3 months and 6 months with a neurointerventionalist. At every clinical follow-up, functional outcome was assessed using the modified Rankin scale (mRS); scores ranging from 0 to 2 were regarded as favourable. DSA was the primary radiological follow-up examination method and was performed at 6 months. Aneurysm occlusion was graded using the Modified Raymond-Roy Occlusion Classification (MRRC) on the final control run and on follow-up angiograms [16].

Neurological complications were defined as the composite of the following complications: spontaneous aneurysm rupture, ipsilateral intracranial haemorrhage, ischaemic stroke, parent artery stenosis, and cranial neuropathy. We defined the degree of ischaemic stroke according to changes in the National Institutes of Health Stroke Scale (NIHSS) score [17–19]: minor neurological stroke was defined as an NIHSS score change ≤ 4, lasting less than 7 days, with confirmatory imaging; transient ischaemia attacks (TIAs) were defined as transient neurological deficits without confirmatory imaging; and major ischaemic stroke was defined as changes in the National Institutes of Health Stroke Scale (NIHSS) score > 4, lasting more than 7 days, with confirmatory imaging [20]. Poor functional outcomes were confirmed by a poorer modified Rankin scale (mRS) score [19, 21], which we defined as an early or late postoperative mRS score that is worse than the preoperative mRS score.

### Statistical analysis

Univariate analysis of discrete data was performed using the chi square test or Fisher’s exact test. T tests were conducted to determine differences between parametric dependent variables. For continuous data with deviation from normality, including analysis of scores or ranked data, nonparametric tests were utilized. Univariate analysis was used to determine whether the covariates predicted the dependent variable, which were neurological complications. Interactions and confounding variables were assessed through stratification and relevant expansion of covariates. Predictive factors identified in the univariate analysis (*P* < 0.05) were entered into the multivariate logistic regression analysis. The risk factors determined by univariate analysis (*P* < 0.05) were also entered into the multivariate logistic regression analysis. *P* values < 0.05 were considered to indicate statistical significance, and all the statistical analyses were conducted with SPSS (IBM Corp., Armonk, NY, USA; Version 25).

## Results

### Baseline characteristics

The demographic and baseline characteristics are presented in Table 1. A total of 522 patients with 533 aneurysms were treated using the Neuroform Atlas Stent. The mean age was 58.9 ± 9.9 years, and 341 (65.3%) patients were female. The comorbidities included hypertension in 337 patients (64.6%), diabetes in 83 patients (15.9%), hyperlipidaemia in 150 patients (28.7%), cerebral infarction in 89 patients (17.0%), cardiac disease in 43 patients (8.2%), peripheral venous thrombosis in 4 patients (0.8%) and renal insufficiency in 6 patients (1.1%). A total of 238 (45.6%) aneurysms were symptomatic, 188 (36.0%) were found incidentally, and 96 (18.4%) caused SAH. Twenty-five (4.8%) aneurysms were treated by clipping or coiling.

**Table 1:** Baseline characteristics of 522 patients treated with the Neuroform Atlas Stent

Characteristic	Frequency (*N* = 522)
**Total number of aneurysms treated with the Neuroform Atlas Stent**	533
**Patients with multiple aneurysms**	157 (30.0%)
**Female**	341 (65.3%)
**Age(years)**	58.9 ± 9.9
**Comorbidities**	
Hypertension	337 (64.6%)
Diabetes	83 (15.9%)
Hyperlipidaemia	150 (28.7%)
Cerebral infarction	89 (17.0%)
Cardiac disease	43(8.2%)
Peripheral venous thrombosis	4 (0.8%)
Renal insufficiency	6(1.1%)
**Smoking**	
Never	402(77.0%)
Previous	55 (10.5%)
Current	65(12.5%)
**Alcohol abuse**	
Never	448 (85.8%)
Previous	35 (6.7%)
Current	39 (7.5%)
**Presentation**	
Incidental	188 (36.0%)
Symptomatic	238(45.6%)
Current SAH	96 (18.4%)
**Hunt-Hess Grade**	
**I-II**	72/96 (75%)
**III-IV**	24/96 (25%)
**Previous treatment(coiling/stent)**	25 (4.8%)
**CYP2C19 genotyping**^**a**^	362 (69.3%)
Fast metabolism	163 (45.0%)
Intermediate metabolism	152 (42.0%)
Slow metabolism	47 (13%)
**Preoperative coagulation test**^**b**^	
Prothrombin time(s)	11.09 ± 1.20
International standard ratio	0.98 ± 0.11
**Platelet aggregation test**^**c**^	
Collagen arachidonic acid (%)	10.01 ± 7.74
Adenosine diphosphate (%)	34.20 ± 13.99
**Homocysteine**^**d**^**(μmol/L)**	12.57 ± 5.65

Data are shown as n (%) or the mean ± SD

^a^153 patients did not have CYP2C19 genotyping reported

^b^12 patients did not have preoperative blood coagulation reported

^c^121 patients did not have platelet aggregation test reported

^d^121 patients did not have homocysteine reported

### Patient characteristics

Aneurysm characteristics are presented in Table 2. The mean aneurysm size, neck size, and parent artery diameter were 5.45 ± 2.69 mm, 4.04 ± 1.66 mm, and 2.66 ± 0.72 mm, respectively. The size ratio, aspect ratio and height/width ratio of the aneurysms were 1.71 ± 0.99, 1.12 ± 0.45 and 0.97 ± 0.34, respectively. The morphology of the aneurysms was saccular in 395 (74.1%) patients and nonsaccular in 138 (25.9%). The majority of occlusions were located in the anterior circulation (proximal 152 (28.5%) and distal 304 (57.1%)), and 77 (14.4%) were located in the posterior circulation, including 57 (10.6%) located in the vertebral-basilar artery and 20 (3.8%) in the vertebral artery and other posterior circulation.

**Table 2:** Aneurysm characteristics (*N* = 533)

Characteristic	Frequency
**Aneurysm morphologies**	
Average aneurysm size (maximum aneurysm length,mm)	5.45 ± 2.69
Average neck size(mm)	4.04 ± 1.66
Average width size(mm)	4.69 ± 2.30
Average height size (mm)	4.34 ± 2.40
Average parent artery diameter(mm)	2.66 ± 0.72
Size ratio	1.71 ± 0.99
Aspect ratio	1.12 ± 0.45
Height/width ratio	0.97 ± 0.34
**Aneurysm form**	
Saccular	395 (74.1%)
Non saccular	138 (25.9%)
**Location**	
Anterior circulation	456 (85.6%)
Anterior circulation proximal	152 (28.5%)
Anterior circulation distal	304 (57.1%)
Posterior circulation	77 (14.4%)
Vertebral-Basilar	57 (10.6%)
Other vessels in the posterior circulation^a^	20 (3.8%)

Data is shown as n (%) or the mean ± SD

^a^Other arteries location on posterior circulation including the posterior cerebral artery, posterior inferior cerebellar artery, anterior inferior cerebellar artery and the superior cerebellar artery

### Treatment details and angiographic and clinical outcomes

**Fig. 2:**
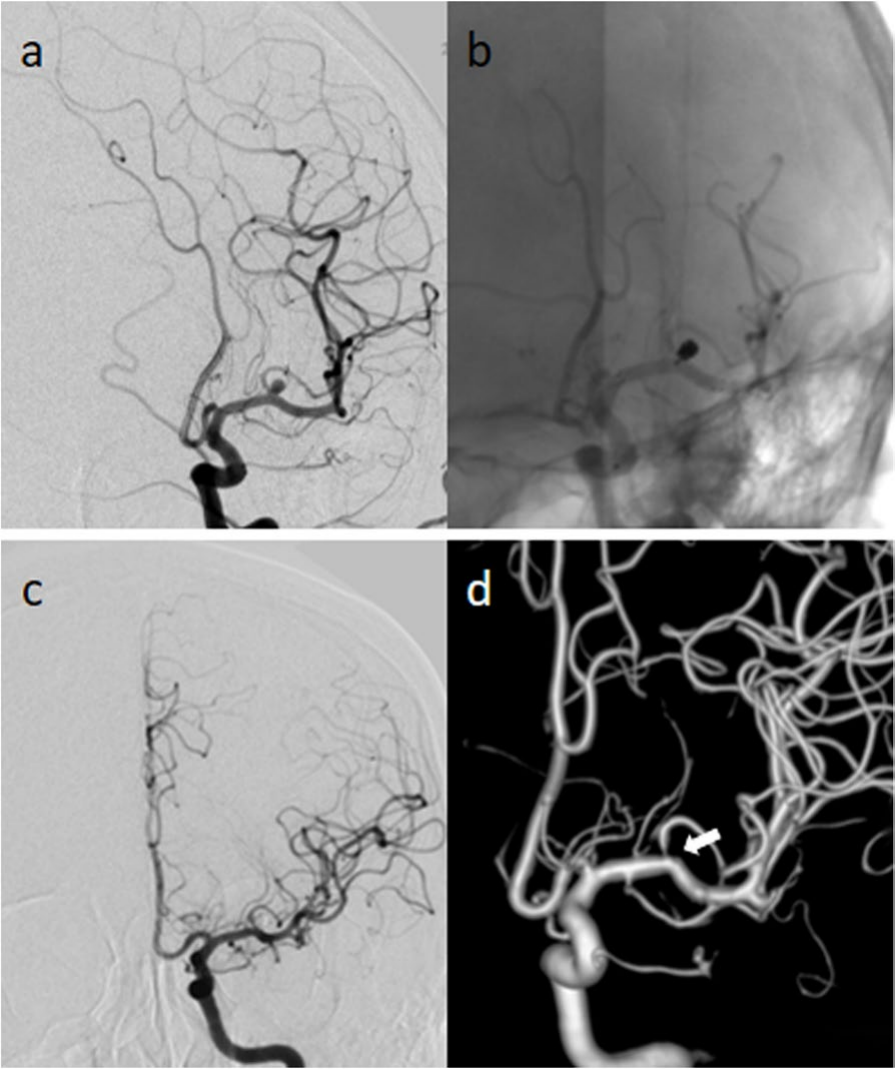
Angiographic images of a left MCA bifurcation aneurysm that was treated with a Neuroform Atlas stent. **a** Positive preoperative DSA image of the left MCA bifurcation aneurysm; **b**, positive postoperative DSA image of the left MCA bifurcation aneurysm; **c**, **d**, positive follow-up DSA image showing complete aneurysm occlusion (arrows)

Treatment details and angiographic and clinical outcomes are presented in Table 3. The diameter and length of the Atlas stent were 3.37 ± 0.55 mm and 18.34 ± 3.55 mm, respectively. A total of 21 (3.9%) aneurysms were treated with multiple NeuroForm Atlas stents, and 512 (96.1%) aneurysms were treated with a single NeuroForm Atlas stent. Only one aneurysm (0.2%) required a single Atlas stent. There were no aneurysms that were clipped at the same institution in our study. Thirty-three (6.2%) patients had preoperative stenosis of the parent artery. The mean procedure duration was 152.83 ± 42.40 min. Coil protrusion into the parent arteries was observed in 18 (3.4%) of aneurysms after atlas stent treatment. At the last follow-up for each patient, the total occlusion rate was 74.5% (397/533). Before Atlas stent implantation, the mRS score was 0–2 for 502 (96.2%) and 3–6 for 20 (3.8%); at the final follow-up, the mRS score was 0–2 for 483 (92.5%) and 3–6 for 39 (7.5%). An illustrative case is shown in Fig. 2.

**Table 3:** Treatment details of 533 aneurysms

Procedural characteristics	Frequency (*N* = 533 aneurysms)
**NeuroForm Atlas Stent size**	
Diameter (mm)	3.37 ± 0.55
Length (mm)	18.34 ± 3.55
**Multiple NeuroForm Atlas Stent used**	21 (3.9%)
**Single NeuroForm Atlas Stent used**	512 (96.1%)
**Blood thinners**	
Preoperative aspirin/clopidogrel > 3 days	439/522 (84.1%)
Preoperative statin > 7 days	111/522 (21.3%)
Preoperative anticoagulant (Warfarin/ Rivaroxaban) > 7 days	5/522 (1.0%)
**Pre-operation parent artery stenosis**	33 (6.2%)
**Coil protrusion**	18 (3.4%)
**Treatment**	
Stent-assisted coiling	532 (99.8%)
Stent	1 (0.2%)
**Aneurysm occlusion status**^**a**^** (Immediate postoperative)**	
Complete occlusion	397 (74.5%)
Incomplete occlusion	136 (25.5%)
**Procedure duration(mins)**^**b**^	152.83 ± 42.40
**Clinical outcomes**	
MRS score	
Preoperative mRS score	
0–2	502/522 (96.2%)
3–6	20/522 (3.8%)
Postoperative mRS score (< 30 d)	
0–2	483/522 (92.5%)
3–6	39/522 (7.5%)

Data are shown as n (%), n/N (%), or the mean ± SD

^*^ The total number of NeuroForm Atlas Stents used was 554

^a^ Records of operative duration were missing for 81 patients

^b^Each Aneurysm was evaluated using the Raymond–Roy occlusion classification (RROC) and dichotomized as occluded (I) or residual (II/III)

### Morbidity, mortality, and complication rates and functional outcome

Table 4 presents the study outcomes. There were 36 patients lost to clinical follow-up. Major ischaemic stroke occurred in 2.1% (11/522) of patients in the early postoperative period and in 0.2% (1/486) during the follow-up period. Minor stroke and/or TIA occurred in 2.9% (15/522) of patients in the early postoperative period and in 0.8% (4/486) of patients during the follow-up period. DAR occurred in 2.3% (12/522) of patients in the early postoperative period and in 0.2% (1/486) during the follow-up period. The rate of distal intraparenchymal haemorrhage (DIPH) was 0.2% (1/522) in the early postoperative period and 0% during the follow-up period. Neurologic morbidity occurred in 7.3% (38/522) of patients during the early postoperative period and in 1.2% (6/486) of patients during the follow-up period. Acute stent thrombosis occurred in 2.3% of patients in the early postoperative period (12/522) and in 0.0% of patients during the follow-up period. Encephalopathy caused by contrast agent occurred in 0.6% (3/522) of patients in the early postoperative period and in 0% of patients during the follow-up period. The mortality rate was 0.6% (3/522) in the early postoperative period and 0.2% (1/486) during the follow-up period. Poor functional outcome (worse mRS score) was observed in 5.4% (28/522) and 0.8% (4/486) of patients in the early postoperative and follow-up periods, respectively.

**Table 4:** Morbidity, mortality, complications, and functional outcomes in the postoperative period

Variable	Early postoperative period (< 30 days) (*N* = 522)	Follow-up period (3–17 months) (*N* = 486)^a^
Major ischaemic stroke	11 (2.1%)	1(0.2%)
TIA/minor stroke	15 (2.9%)	4(0.8%)
DAR	12 (2.3%)	1(0.2%)
DIPH	1 (0.2%)	0
Neurologic morbidity^b^	38(7.3%)	6(1.2%)
Stent acute thrombosis	12 (2.3%)	0
Contrast Encephalopathy	3 (0.6%)	0
Mortality	3(0.6%)	1 (0.2%)
Artery stenosis	2(0.4%)	2(0.4%)
Epilepsy	1 (0.2%)	1 (0.2%)
MRS score deterioration^c^	28 (5.4%)	4(0.8%)

Data are shown as n (%)

^a^There were 36 patients lost to clinical follow-up

^b^ Numbers do not sum across categories and subcategories because some patients experienced > 1 event

^**c**^MRS score deterioration = Postoperative mRS score (< 30 d) /(3–17 mo)- Preoperative mRS score

### Predictors of stroke

Multivariate analyses for predictors of ischaemic stroke, TIA, and minor stroke during the entire study duration are presented in Supplementary Table 1. Cerebral infarction (OR = 4.324, *P* = 0.001), Hunt–Hess grade (3–5) (OR = 2.741, *P* = 0.001), procedure duration (OR = 1.011, *P* = 0.005), stent length (OR = 1.197, *P* = 0.002) and coil protrusion (OR = 6.177, *P* = 0.004) were found to be independent predictors of ischaemic stroke.

### Predictors of delayed aneurysmal rupture (DAR)

According to multivariate analysis, cardiac disease (OR = 6.575, *P* = 0.004) and incomplete postoperative occlusion (OR = 5.078, *P* = 0.006) were found to be independent predictors of DAR (Supplementary Table 2).

### Predictors of neurologic morbidity

According to multivariate analysis, cerebral infarction (OR = 2.869, *P* = 0.009), Hunt–Hess grade (3–5) (OR = 2.408, *P* = 0.001), procedure duration (OR = 1.010, *P* = 0.003), stent length (OR = 1.134, *P* = 0.010) and coil protrusion (OR = 3.941, *P* = 0.025) were found to be independent predictors of neurologic morbidity (Supplementary Table 3).

### Predictors of acute stent thrombosis in the early postoperative period

According to multivariate analysis, preoperative anticoagulant therapy (warfarin/rivaroxaban) > 7 days (OR = 0.101, *P* < 0.0001) was protective against the formation of acute in-stent thrombosis. Size ratio (OR = 1.596, *P* = 0.005) and coil protrusion (OR = 15.060, *P* = 0.001) were found to be independent predictors of acute stent thrombosis in the early postoperative period (Supplementary Table 4).

### Predictors of poor functional outcome in the early postoperative period

**Fig. 3:**
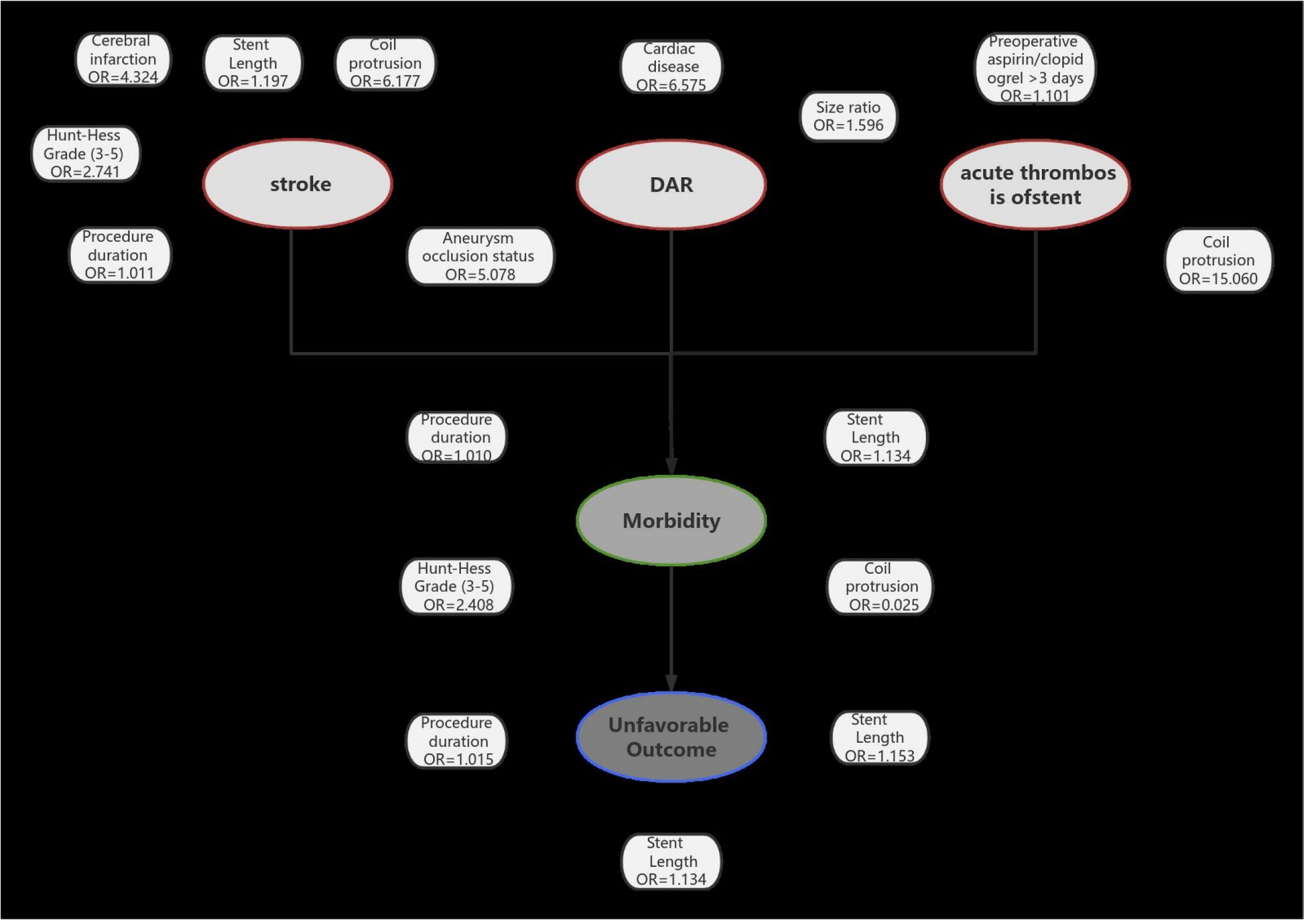
A diagram showing the predictors of complications, morbidity, and unfavourable outcomes

According to the multivariate analysis, the independent predictors of poor functional outcome included procedure duration (OR = 1.789, *P* = 0.020), stent length (OR = 1.153, *P* = 0.021) and coil protrusion (OR = 6.451, *P* = 0.004) (Supplementary Table 5). The results interpretation chart is shown on Fig. 3.

## Discussion

Our study revealed that Neuroform Atlas stent implantation for the treatment of aneurysms is safe, with acceptable rates of morbidity and mortality. The neurological morbidity rate was 7.3% in the early postoperative period and 1.2% during the follow-up period. The complications that occurred in the early postoperative period were as follows: ischaemic stroke (5.0%), DAR (2.3%), DIPH (0.2%), acute stent thrombosis (2.3%), and mortality (0.6%). During the follow-up period, the complications were as follows: ischaemic stroke (1.0%), DAR (0.2%), DIPH (0%), acute stent thrombosis (0%), and mortality (0.2%). Approximately 86% of the major adverse events, particularly ischaemic stroke, DIPH and DAR, occurred in the early postoperative period in our study. Finally, the percentage of patients whose mRS scores deteriorated during the early postoperative period and during the follow-up period was 5.4% and 0.8%, respectively. Importantly, this study identified procedure duration and stent length as novel independent predictors of SCA-related ischaemic stroke, neurological morbidity and mRS score deterioration in the postoperative period.

### Ischaemic stroke and acute stent thrombosis

The rates of major ischaemic stroke complications (2.1%) and stent thrombosis (2.3%) in our study were comparable to the rates of periprocedural major ischaemic stroke (2.9%) and stent thrombosis (1.1%) reported in a systematic review and meta-analysis by Lynch et al. [22] Similarly, a multicentre analysis of 128 aneurysms in 128 patients revealed that the rate of symptomatic thromboembolic stroke was 2.3% [23]. The rate of thromboembolic events associated with the NeuroForm Atlas stent was lower than that associated with the LVIS device (4.9%), Neuroform (6.7%) and Enterprise (5.9%) stents according to a meta-analysis [24, 25]. A study comparing the complication rates of Pipeline and Atlas stent implantation for aneurysms showed that the midterm complication rates of the PED and Atlas SAC for the treatment of ICA aneurysms were similar (5.6% vs. 11.2%, *P *= 0.177) [26]. However, there are few studies focused on identifying the risk factors of ischaemic stroke and stent thrombosis following Atlas stent implantation. In our study, the majority of major ischaemic strokes occurred within the early postoperative period; cerebral infarction, Hunt–Hess grade (3–5), procedure duration, stent length and coil protrusion were found to be independent predictors of ischaemic stroke. Preoperative anticoagulant therapy (warfarin/rivaroxaban) > 7 days was protective against the formation of acute in-stent thrombosis. Size ratio and coil protrusion were found to be independent predictors of acute stent thrombosis in the early postoperative period. Russo et al. [27] reported that the formation of stent thrombosis mostly occurs during the postoperative period, which may be related to the deposition of protein membranes after stent implantation [28]. Therefore, it is important to prioritize early antiplatelet therapy. Prolonged and multiple operative procedures may result in endothelial damage due to repeated stent adjustments, which can increase the risk of ischaemic stroke [15]. Patients with subarachnoid haemorrhage (SAH) are in a hypercoagulable state and are more susceptible to thromboembolism [29]. Longer stents have the potential to obstruct the collateral circulation, resulting in reduced or blocked blood flow and leading to ischaemic stroke. Additionally, coil herniation into the parent artery can impede blood flow, contributing to ischaemic stroke [30].

### DAR and DIPH

Lynch et al. [22] reported an incidence of 1.4% for intraprocedural rupture or vessel dissection and 1.0% for periprocedural or early haemorrhage. A study in China reported that the incidence of haemorrhagic complications was 1.4% [26]. According to a multicentre postmarket analysis, 2 patients (1.6%) experienced haemorrhagic complications, with one experiencing complications during the procedure and the second experiencing complications during the postoperative follow-up period [23].

In comparison, our study revealed a slightly higher rate of DAR (2.5%), with 92% of DARs occurring in the early postoperative period. Cardiac disease and incomplete occlusion immediately after the procedure were identified as independent predictors of DAR during the early postoperative period. In our study, only one patient experienced DIPH during the early postoperative period. Incomplete occlusion immediately after the procedure may result in acute thrombosis within the aneurysm. Kulcsar et al. [31] hypothesized that the rapid formation of an intraluminal thrombus could trigger protease secretion, de-endothelialization, and leukocyte infiltration of the aneurysm wall, weakening the wall integrity and eventually leading to aneurysm rupture.

### Functional outcomes

Sweid et al. [16] reported that 91.8% of patients had a favourable clinical outcome (mRS score 0–2), with a mortality rate of 1.4%. Hanel et al. [32] reported 2 unrelated deaths occurring beyond 30 days after the procedure, and 84.4% (27/32) of patients had a modified Rankin score ranging from 0 to 2 one year after the procedure. In our study, the mortality rate during the early postoperative period was 0.6% and 0.2% during the follow-up period. Poor functional outcome (MRS score deterioration) was observed in 5.4% and 0.8% of patients during the early postoperative and follow-up periods, respectively, which aligns with previously reported findings. Additionally, procedure duration, stent length, and coil protrusion were found to be independent predictors of poor functional outcome, which are associated with complications, including ischaemic stroke.

Overall, procedure duration and stent length were found to be novel independent predictors of SCA-related ischaemic stroke, neurologic morbidity, and mRS score deterioration during the postoperative period. Our study reports the predictive factors for evaluating the risk of complications following Atlas stent implantation for intracranial aneurysms in Chinese patients.

### Limitations

The limitations of this study include its single-centre, retrospective design, which introduces potential biases. Additionally, the relatively short follow-up duration may have resulted in an underestimation of the complication rate.

## Conclusions

The incidence of SCA (stent-assisted coiling)-related complications with the Neuroform Atlas stent in the cohort of Chinese patients in this study was similar to that in the Western population. We found that procedure duration and stent length were novel independent predictors of SCA-related ischaemic stroke, neurologic morbidity and mRS score deterioration in the postoperative period in a Chinese population.

### Supplementary Information

Supplementary Material 1.

## Data Availability

Data is provided within the manuscript and supplementary information files.

## Abbreviations

SCA: Stent-assisted coiling
mRS: Modified Rankin scale
SR: Size ratio
AR: Aspect ratio
HW: Height/width ratio
DAR: Delayed aneurysm rupture
DIPH: Distal intraparenchymal haemorrhage
TIA: Transient ischaemic attack
SAH: Subarachnoid haemorrhage
DSA: Digital subtraction angiography
OR: Odds ratio
