# Supplementary material for: Neuroform atlas stent treatment for 533 intracranial aneurysms in a large Chinese cohort: complication risk factor analysis
Source: BMC Neurol. 2024 Jun 10;24:195. doi: 10.1186/s12883-024-03695-z (PMC11163780; doi:10.1186/s12883-024-03695-z)
Supplement: Supplementary file 1 — Supplementary Material 1. [file 12883_2024_3695_MOESM1_ESM.docx]

**Neuroform Atlas Stent for Intracranial Aneurysms in a Large Chinese Cohort: Complications Risk Factor Analysis**

**Online only supplementary material**

**Supplementary Table 1.** Univariate and multivariate analysis for predictors of ischaemic stroke

|  | **Postoperative period (n=31)** | | |
| --- | --- | --- | --- |
| **Variable** | **Univariate analysis** | **Multivariate analysis** |  |
| **Female** | 0.956 [0.448-2.041] 0.908 |  |  |
| **Age** | 1.005 [0.968-1.042] 0.808 |  |  |
| **Hypertension** | 1.390 [0.627-3.804] 0.418 |  |  |
| **Diabetes** | **2.293 [1.017-5.170] 0.045** |  |  |
| **Hyperlipidaemia** | 1.617[0.765-3.418] 0. 208 |  |  |
| **Cardiac disease** | 1.237 [0.360-4.249] 0.735 |  |  |
| **Cerebral infarction** | **2.550 [1.157-5.620] 0.020** | **4.324 [1.782-10.491]0.001** |  |
| **Peripheral venous thrombosis** | ― |  |  |
| **Smoking** |  |  |  |
| Never | 1 |  |  |
| Previous | 0.623 [0.143-2.718] 0.529 |  |  |
| Current | 1.655 [0.648-4.229] 0.293 |  |  |
| **Alcohol abuse** |  |  |  |
| Never | 1 |  |  |
| Previous | 0.935 [0.213-4.098] 0.929 |  |  |
| Current | 0.815 [0.187-3.555] 0.785 |  |  |
| **Previous treatment(coiling/stent)** | 1.374 [0.309-6.097] 0.676 |  |  |
| **Preoperative aspirin/clopidogrel >3 days** | 0.621 [0.259-1.492] 0.287 |  |  |
| **Preoperative anticoagulant (Warfarin/ Rivaroxaban) >7days** | ― |  |  |
| **Preoperative statin >7days** | 1.492 [0.668-3.335] 0.329 |  |  |
| **Preoperative coagulation test** |  |  |  |
| [Prothrombin time](https://www.baidu.com/link?url=55as5c2j0bxhXux60fn_jGEKV6gMhUHwt4QGo2VuZEXjjGd0rGt1vWpNbXr84H8VUmyZGd7ccxMwceUN7QL7yq&wd=&eqid=8e3b61e2000c197600000003629e2ac7) | 0.936 [0.622-1.408] 0.749 |  |  |
| International standard ratio | 0.649 [0.011-37.441]0.835 |  |  |
| **Platelet aggregation test** |  |  |  |
| Collagen arachidonic acid | 1.009 [0.971-1.049] 0.633 |  |  |
| Adenosine diphosphate | 1.004 [0.978-1.030] 0.780 |  |  |
| **Homocysteine** | 1.008 [0.950-1.069] 0.804 |  |  |
| **Hunt-Hess Grade (3-5)** | **2.090[1.238-3.527] 0.006** | **2.741[1.539-4.882] 0.001** |  |
| **Aneurysm Location(Anterior)** | **0.458[0.197-1.065] 0.070** |  |  |
| **Aneurysm form(Non saccular)** | **1.885 [0.890-3.991] 0.098** |  |  |
| **Multiple aneurysms** | 0.684 [0.289-1.623] 0.389 |  |  |
| **Average aneurysm size (maximum aneurysm length)** | 1.053[0.943-1.176] 0.360 |  |  |
| **Average neck size** | 1.023[0.827-1.266] 0.831 |  |  |
| **Average width size** | 1.066 [0.928-1.224] 0.369 |  |  |
| **Average height size** | **1.076 [0.963-1.201] 0.194** |  |  |
| **Average parent artery diameter** | 1.050[0.640-1.721] 0.848 |  |  |
| **Size ratio** | **1.257 [0.965-1.638] 0.090** |  |  |
| **Aspect ratio** | **1.697 [0.859-3.352] 0.128** |  |  |
| **Height/width ratio** | 1.194 [0.431-3.307] 0.733 |  |  |
| **Pre-operation parent artery stenosis** | 1.686 [0.485-5.863] 0.412 |  |  |
| **Procedure duration** | **1.011 [1.004-1.018] 0.002** | **1.011 [1.003-1.018] 0.005** |  |
| **Multiple NeuroForm Atlas Stent used** | 0.727 [0.095-5.580] 0.759 |  |  |
| **Stent diameter** | 0.782 [0.386-1.585] 0.496 |  |  |
| **Stent Length** | **1.164 [1.052-1.287] 0.003** | **1.197 [1.070-1.340] 0.002** |  |
| **Treatment** | ― |  |  |
| **Aneurysm occlusion status**  **(Immediate postoperative)** | 1.421 [0.652-3.099] 0.377 |  |  |
| **Coil protrusion** | **5.164 [1.592-16.752]0.006** | **6.177 [1.786-21.362]0.004** |  |

**Supplementary Table 2.** Univariate and multivariate analysis for predictors of delayed aneurysmal rupture（DAR）in the early postoperative period

|  | **Postoperative early period (n=12)** | | |
| --- | --- | --- | --- |
| **Variable** | **Univariate analysis** | **Multivariate analysis** |  |
| **Female** | **2.962 [0.649-13.505]0.161** |  |  |
| **Age** | 0.975[0.925-1.028] 0.344 |  |  |
| **Hypertension** | 0.891 [0.287-2.763] 0.842 |  |  |
| **Diabetes** | 0.957[0.208-4.398] 0.955 |  |  |
| **Hyperlipidaemia** | 0.444[0.097-2.028] 0. 295 |  |  |
| **Cardiac disease** | **5.481 [1.615-18.607]0.006** | **6.575 [1.821-23.736]0.004** |  |
| **Cerebral infarction** | 0.409 [0.053-3.187] 0.393 |  |  |
| **Peripheral venous thrombosis** | ― |  |  |
| **Smoking** | ― |  |  |
| **Alcohol abuse** | ― |  |  |
| **Previous treatment(coiling/stent)** | ― |  |  |
| **Preoperative aspirin/clopidogrel >3 days** | **0.409 [0.123-1.360] 0.145** |  |  |
| **Preoperative anticoagulant (Warfarin/Rivaroxaban) >7days** | ― |  |  |
| **Preoperative statin >7days** | 1.068 [0.289-3.947] 0.921 |  |  |
| **Preoperative coagulation test** |  |  |  |
| [Prothrombin time](https://www.baidu.com/link?url=55as5c2j0bxhXux60fn_jGEKV6gMhUHwt4QGo2VuZEXjjGd0rGt1vWpNbXr84H8VUmyZGd7ccxMwceUN7QL7yq&wd=&eqid=8e3b61e2000c197600000003629e2ac7) | 0.741 [0.332-1.654] 0.465 |  |  |
| International standard ratio | 0.025 [0.000004-152.057] 0.407 |  |  |
| **Platelet aggregation test** |  |  |  |
| Collagen arachidonic acid | 0.942 [0.769-1.154] 0.563 |  |  |
| Adenosine diphosphate | 0.991 [0.952-1.032] 0.679 |  |  |
| **Homocysteine** | 0.975 [0.863-1.102] 0.686 |  |  |
| **Hunt-Hess Grade** | 1.317 [0.521-3.326] 0.560 |  |  |
| **Aneurysm Location(Anterior)** | **0.367 [0.110-1.224] 0.103** |  |  |
| **Aneurysm form** | 1.819 [0.585-5.656] 0.302 |  |  |
| **Multiple aneurysms** | 1.513 [0.487-4.699] 0.474 |  |  |
| **Average aneurysm size (maximum aneurysm length)** | **1.106[0.973-1.256] 0.124** |  |  |
| **Average neck size** | 1.136[0.857-1.504] 0.375 |  |  |
| **Average width size** | 1.112[0.923-1.340] 0.264 |  |  |
| **Average height size** | **1.105 [0.967-1.263] 0.142** |  |  |
| **Average parent artery diameter** | 0.846[0.381-1.879] 0.682 |  |  |
| **Size ratio** | 1.242 [0.858-1.796] 0.250 |  |  |
| **Aspect ratio** | 1.246 [0.404-3.840] 0.702 |  |  |
| **Height/width ratio** | 1.912 [0.523-6.992] 0.328 |  |  |
| **Pre-operation parent artery stenosis** | **2.868 [0.609-13.509]0.183** |  |  |
| **Procedure duration** | **1.010[1.000-1.020] 0.055** |  |  |
| **Multiple NeuroForm Atlas Stent used** | ― |  |  |
| **Stent diameter** | 1.337 [0.517-3.459] 0.549 |  |  |
| **Stent Length** | 1.084 [0.934-1.259] 0.288 |  |  |
| **Treatment** | ― |  |  |
| **Aneurysm occlusion status (Incomplete occlusion of immediate postoperative)** | **4.900[1.575-15.245] 0.006** | **5.078 [1.596-16.160]0.006** |  |
| **Coil protrusion** | ― |  |  |

Data is shown as the odds ratio (95% CI) P-value.

“―” indicates that the listed complication did not occur in any of the patients.

**Supplementary Table 3.** Univariate and multivariate analysis for predictors of neurologic morbidity in the postoperative period

|  | **Postoperative period (n=43)** | | |
| --- | --- | --- | --- |
| **Variable** | **Univariate analysis** | **Multivariate analysis** |  |
| **Female** | 1.147 [0.579-2.273] 0.693 |  |  |
| **Age** | 0.996[0.964-1.028] 0.793 |  |  |
| **Hypertension** | 1.221 [0.617-2.417] 0.567 |  |  |
| **Diabetes** | **2.071[0.994-4.314] 0.052** |  |  |
| **Hyperlipidaemia** | 1.167[0.588-2.319] 0.659 |  |  |
| **Cardiac disease** | **2.608[1.080-6.296] 0.033** |  |  |
| **Cerebral infarction** | **1.946[0.936-4.046] 0.075** | **2.869[1.297-6.346] 0.009** |  |
| **Peripheral venous thrombosis** | ― |  |  |
| **Smoking** |  |  |  |
| Never | 1 |  |  |
| Previous | 0.940 [0.319-2.772] 0.911 |  |  |
| Current | 1.203[0.482-3.003] 0.693 |  |  |
| **Alcohol abuse** |  |  |  |
| Never | 1 |  |  |
| Previous | 1.061 [0.310-3.628] 0.925 |  |  |
| Current | 0.598 [0.139-2.579] 0.491 |  |  |
| **Previous treatment(coiling/stent)** | 1.000 [0.228-4.389] 1.000 |  |  |
| **Preoperative aspirin/clopidogrel >3 days** | **0.475 [0.228-0.990] 0.047** |  |  |
| **Preoperative anticoagulant (Warfarin/ Rivaroxaban) >7 days** | ― |  |  |
| **Preoperative statin >7days** | 1.335 [0.648-2.753] 0.434 |  |  |
| **Preoperative coagulation test** |  |  |  |
| [Prothrombin time](https://www.baidu.com/link?url=55as5c2j0bxhXux60fn_jGEKV6gMhUHwt4QGo2VuZEXjjGd0rGt1vWpNbXr84H8VUmyZGd7ccxMwceUN7QL7yq&wd=&eqid=8e3b61e2000c197600000003629e2ac7) | 0.941 [0.663-1.336] 0.733 |  |  |
| International standard ratio | 0.508 [0.011-23.485]0.729 |  |  |
| **Platelet aggregation test** |  |  |  |
| Collagen arachidonic acid | 1.004 [0.967-1.043] 0.834 |  |  |
| Adenosine diphosphate | 0.999 [0.976-1.022] 0.900 |  |  |
| **Homocysteine** | 0.988[0.928-1.052] 0.708 |  |  |
| **Hunt-Hess Grade (3-5)** | **2.042[1.273-3.275] 0.003** | **2.408[1.451-3.994] 0.001** |  |
| **Aneurysm Location**（Anterior） | **0.423 [0.202-0.804] 0.022** |  |  |
| **Aneurysm form** (Non saccular) | **2.172 [1.129-4.178] 0.020** |  |  |
| **Multiple aneurysms** | 0.757 [0.362-1.584] 0.460 |  |  |
| **Average aneurysm size (maximum aneurysm length)** | 1.043[0.942-1.155] 0.421 |  |  |
| **Average neck size** | 1.007[0.832-1.218] 0.946 |  |  |
| **Average width size** | 1.038[0.912-1.181] 0.574 |  |  |
| **Average height size** | 1.064[0.959-1.180] 0.244 |  |  |
| **Average parent artery diameter** | 0.934[0.596-1.462] 0.765 |  |  |
| **Size ratio** | **1.212 [0.944-1.556] 0.131** |  |  |
| **Aspect ratio** | **1.509[0.808-2.819] 0.197** |  |  |
| **Height/width ratio** | 1.350 [0.567-3.214] 0.498 |  |  |
| **Pre-operation parent artery stenosis** | 1.726 [0.576-5.173] 0.330 |  |  |
| **Procedure duration** | **1.011[1.004-1.017] 0.001** | **1.010[1.004-1.017] 0.003** |  |
| **Multiple NeuroForm Atlas Stent used** | 0.534 [0.070-4.066] 0.545 |  |  |
| **Stent diameter** | 0.774 [0.416-1.439] 0.418 |  |  |
| **Stent Length** | **1.124[1.029-1.227] 0.009** | **1.134[1.031-1.247] 0.010** |  |
| **Treatment** | ― |  |  |
| **Aneurysm occlusion status (Incomplete occlusion of immediate postoperative)** | **1.769[0.907-3.449] 0.094** |  |  |
| **Coil protrusion** | **3.691[1.157-11.780] 0.027** | **3.941[1.190-13.051] 0.025** |  |

Data is shown as the odds ratio (95% CI) P-value.

“―” indicates that the listed complication did not occur in any of the patients.

**Supplementary Table 4.** Univariate and multivariate analysis for predictors of Stent acute thrombosis in the early postoperative period

|  | **Postoperative** **early period (n=12)** | |
| --- | --- | --- |
| **Variable** | **Univariate analysis** | **Multivariate analysis** |
| **Female** | 0.733 [0.229-2.342] 0.600 |  |
| **Age** | 1.020[0.961-1.084] 0.511 |  |
| **Hypertension** | 0.551 [0.175-1.731] 0.307 |  |
| **Diabetes** | 0.473[0.060-3.712] 0.476 |  |
| **Hyperlipidaemia** | 0.824[0.220-3.087] 0. 774 |  |
| **Cardiac disease** | ― |  |
| **Cerebral infarction** | 1.686 [0.447-6.356] 0.440 |  |
| **Peripheral venous thrombosis** | ― |  |
| **Smoking** |  |  |
| Never | 1 |  |
| Previous | 0.914 [0.112-7.445] 0.933 |  |
| Current | 2.355 [0.609-9.112] 0.215 |  |
| **Alcohol abuse** |  |  |
| Never | 1 |  |
| Previous | 1.274 [0.159-10.243]0.820 |  |
| Current | 1.115 [0.139-8.933] 0.918 |  |
| **Previous treatment(coiling/stent)** | ― |  |
| **Preoperative aspirin/clopidogrel >3 days** | **0.124 [0.038-0.400] p＜0.0001** | **0.101[0.028-0.360] p＜0.0001** |
| **Preoperative anticoagulant (Warfarin/ Rivaroxaban) >7 days** | ― |  |
| **Preoperative statin >7days** | 0.706 [0.153-3.568] 0.656 |  |
| **Preoperative coagulation test** |  |  |
| [Prothrombin time](https://www.baidu.com/link?url=55as5c2j0bxhXux60fn_jGEKV6gMhUHwt4QGo2VuZEXjjGd0rGt1vWpNbXr84H8VUmyZGd7ccxMwceUN7QL7yq&wd=&eqid=8e3b61e2000c197600000003629e2ac7) | 0.836 [0.380-1.837] 0.655 |  |
| International standard ratio | 0.163 [0.000038-704.338] 0.671 |  |
| **Platelet aggregation test** |  |  |
| Collagen arachidonic acid | **1.032 [0.996-1.069] 0.080** |  |
| Adenosine diphosphate | 1.015 [0.975-1.056] 0.470 |  |
| **Homocysteine** | 0.979 [0.865-1.108] 0.734 |  |
| **Hunt-Hess Grade (3-5)** | **3.639 [1.802-7.349] p＜0.001** |  |
| **Aneurysm Location** | 0.841 [0.181-3.913] 0.825 |  |
| **Aneurysm form (**Non saccular**)** | **6.015 [1.782-20.304] 0.004** |  |
| **Multiple aneurysms** | 1.734 [0.542-5.548] 0.354 |  |
| **Average aneurysm size (maximum aneurysm length)** | **1.138[1.011-1.282] 0.032** |  |
| **Average neck size** | 1.145[0.858-1.528] 0.359 |  |
| **Average width size** | **1.149[0.961-1.375] 0.128** |  |
| **Average height size** | **1.145[1.013-1.294] 0.031** |  |
| **Average parent artery diameter** | 1.061[0.488-2.308] 0.881 |  |
| **Size ratio** | **1.489 [1.093-2.029] 0.012** | **1.596 [1.153-2.210] 0.005** |
| **Aspect ratio** | **2.343[0.932-5.888] 0.070** |  |
| **Height/width ratio** | **3.132 [1.018-9.636] 0.046** |  |
| **Pre-operation parent artery stenosis** | **5.456 [1.404-21.206]0.014** |  |
| **Procedure duration** | **1.011[1.001-1.021] 0.037** |  |
| **Multiple NeuroForm Atlas Stent used** | **4.762[0.981-23.112] 0.053** |  |
| **Stent diameter** | 1.162 [0.421-3.202] 0.772 |  |
| **Stent Length** | 0.972 [0.824-1.146] 0.735 |  |
| **Treatment** | ― |  |
| **Aneurysm occlusion status (Incomplete occlusion of immediate postoperative)** | **4.254[1.327-13.635] 0.015** |  |
| **Coil protrusion** | **11.244 [2.762-45.778] 0.001** | **15.060 [3.117-72.768] 0.001** |

Data is shown as the odds ratio (95% CI) P-value.

“―” indicates that the listed complication did not occur in any of the patients.

**Supplementary Table 5.** Univariate and multivariate analysis for predictors of neurological mRS score deterioration in the early postoperative period

|  | **Postoperative early period (n=28)** | | |
| --- | --- | --- | --- |
| **Variable** | **Univariate analysis** | **Multivariate analysis** |  |
| **Female** | 1.619 [0.675-3.882] 0.280 |  |  |
| **Age** | 0.999[0.961-1.038] 0.945 |  |  |
| **Hypertension** | **2.120 [0.844-5.323] 0.110** |  |  |
| **Diabetes** | **2.223[0.946-5.228] 0.067** |  |  |
| **Hyperlipidaemia** | 1.406[0.634-3.120] 0. 402 |  |  |
| **Cardiac disease** | 1.395[0.404-4.822] 0. 599 |  |  |
| **Cerebral infarction** | **2.094 [0.892-4.917] 0.090** |  |  |
| **Peripheral venous thrombosis** | ― |  |  |
| **Smoking** |  |  |  |
| Never | 1 |  |  |
| Previous | 0.596 [0.137-2.592] 0.490 |  |  |
| Current | 0.495 [0.114-2.114] 0.347 |  |  |
| **Alcohol abuse** |  |  |  |
| Never | 1 |  |  |
| Previous | 1.014 [0.230-4.464] 0.985 |  |  |
| Current | 0.431 [0.057-3.265] 0.415 |  |  |
| **Previous treatment(coiling/stent)** | 0.711 [0.093-5.447] 0.743 |  |  |
| **Preoperative aspirin/clopidogrel >3 days** | 0.670 [0.263-1.705] 0.401 |  |  |
| **Preoperative anticoagulant (Warfarin/ Rivaroxaban) >7days** | ― |  |  |
| **Preoperative statin >7days** | 1.453 [0.623-3.389] 0.387 |  |  |
| **Preoperative coagulation test** |  |  |  |
| [Prothrombin time](https://www.baidu.com/link?url=55as5c2j0bxhXux60fn_jGEKV6gMhUHwt4QGo2VuZEXjjGd0rGt1vWpNbXr84H8VUmyZGd7ccxMwceUN7QL7yq&wd=&eqid=8e3b61e2000c197600000003629e2ac7) | 0.959 [0.648-1.419] 0.834 |  |  |
| International standard ratio | 0.959 [0.025-36.178]0.982 |  |  |
| **Platelet aggregation test** |  |  |  |
| Collagen arachidonic acid | 1.010 [0.971-1.051] 0.628 |  |  |
| Adenosine diphosphate | 1.006 [0.979-1.034] 0.659 |  |  |
| **Homocysteine** | 1.014 [0.956-1.074] 0.649 |  |  |
| **Hunt-Hess Grade (3-5)** | **1.531[0.834-2.809] 0.169** |  |  |
| **Aneurysm Location**（Anterior） | **0.275 [0.122-0.622] 0.002** |  |  |
| **Aneurysm form** (Non saccular) | **0.256 [1.039-4.897] 0.040** |  |  |
| **Multiple aneurysms** | 0.789 [0.328-1.895] 0.596 |  |  |
| **Average aneurysm size (maximum aneurysm length)** | 1.072[0.963-1.194] 0.205 |  |  |
| **Average neck size** | 1.104[0.900-1.354] 0.344 |  |  |
| **Average width size** | **1.098[0.957-1.258] 0.182** |  |  |
| **Average height size** | **1.087[0.973-1.214] 0.139** |  |  |
| **Average parent artery diameter** | 1.022[0.606-1.723] 0.936 |  |  |
| **Size ratio** | **1.278 [0.976-1.673] 0.074** |  |  |
| **Aspect ratio** | 1.396[0.656-2.970] 0.387 |  |  |
| **Height/width ratio** | 1.182 [0.405-3.450] 0.759 |  |  |
| **Pre-operation parent artery stenosis** | **2.736 [0.890-8.409] 0.079** |  |  |
| **Procedure duration** | **1.016[1.009-1.023] <0.001** | **1.015[1.007-1.022] <0.001** |  |
| **Multiple NeuroForm Atlas Stent used** | ― |  |  |
| **Stent diameter** | 0.774 [0.368-1.629] 0.500 |  |  |
| **Stent Length** | **1.164[1.048-1.294] 0.005** | **1.153[1.022-1.301] 0.021** |  |
| **Treatment** | ― |  |  |
| **Aneurysm occlusion status (Incomplete occlusion of immediate postoperative)** | **2.692[1.246-5.813] 0.012** |  |  |
| **Coil protrusion** | **5.845 [1.788-19.105] 0.003** | **6.451 [1.811-22.978] 0.004** |  |

Data is shown as the odds ratio (95% CI) P-value.

“―” indicates that the listed complication did not occur in any of the patients.
